# Identification of canine norovirus in dogs in South Korea

**DOI:** 10.1186/s12917-018-1723-6

**Published:** 2018-12-21

**Authors:** Kwang-Soo Lyoo, Min-Chul Jung, Sun-Woo Yoon, Hye Kwon Kim, Dae Gwin Jeong

**Affiliations:** 1 Korea Zoonosis Research Institute, Chonbuk National University, Iksan, South Korea; 2 Infectious Disease Research Center, Korea Research Institute of Bioscience and Biotechnology, Daejeon, 305-806 South Korea; 3 University of Science and Technology (UST), Daejeon, South Korea

**Keywords:** Canine norovirus, Dog, Korea

## Abstract

**Background:**

Canine noroviruses (CaNoVs) are classified into genogroups GIV, GVI, and GVII and have been detected in fecal samples from dogs since their first appearance in a dog with enteritis in Italy in 2007. CaNoVs may be a public health concern because pet animals are an integral part of the family and could be a potential reservoir of zoonotic agents. Nonetheless, there was no previous information concerning the epidemiology of CaNoV in South Korea. In the present study, we aimed to detect CaNoV antigens and to investigate serological response against CaNoV in dogs.

**Results:**

In total, 459 fecal samples and 427 sera were collected from small animal clinics and animal shelters housing free-roaming dogs in geographically distinct areas in South Korea. For the detection of CaNoV, RT-PCR was performed using target specific primers, and nucleotide sequences of CaNoV isolates were phylogenetically analyzed. Seroprevalence was performed by ELISA based on P domain protein. CaNoVs were detected in dog fecal samples (14/459, 3.1%) and were phylogenetically classified into the same cluster as previously reported genogroup GIV CaNoVs. Seroprevalence was performed, and 68 (15.9%) of 427 total dog serum samples tested positive for CaNoV IgG antibodies.

**Conclusion:**

This is the first study identifying CaNoV in the South Korean dog population.

## Background

Noroviruses (NoVs) belong to the family *Caliciviridae,* which are non-enveloped viruses approximately 35 nm in diameter with a positive single-strand RNA genome of about 7.7 kb [1]. Human NoVs are responsible for gastroenteritis in humans across all age groups, and typical symptoms of nausea, abdominal pain, fever, vomiting, and diarrhea can be demonstrated in an infected person [2]. The fecal-oral route through contaminated food or water is the major pathway of the viral transmission [3]. Several characteristics of NoVs such as low infectious doses or high resistance to harsh environmental conditions facilitate the spread and infection of the virus [1, 4].

NoVs has been genetically classified into seven major genogroups designated GI through GVII based on sequence analysis of the VP1 protein (ORF2; major capsid protein) [5]. Recently, genotypes are classified by the sequence diversity of the ORF1-ORF2 junction region and the RNA-dependent RNA polymerase (RdRp) region within the ORF1 and VP1 region [5–8]. In humans, three genogroups GI, GII, and GIV are found, and GII strains are the most frequently detected. Additionally, bovine, porcine, and murine NoVs are classified in GIII, GII, and GV, respectively [9].

Canine norovirus (CaNoV) was first identified in a single dog with enteritis in Italy in 2007 [10]. The detection of CaNoVs in fecal samples from dogs was followed in Portugal circa 2007, and then these viruses have been described in dogs from several countries in Europe and Asia [11–14]. A recent prevalence study demonstrated that seropositivity for CaNoV was estimated to be 39% in dog serum samples from 14 different European countries [15]. In addition, it was suggested that CaNoV may infect humans, particularly veterinary workers with potential risk factors for virus exposure. Mesquita et al. reported that they tested serum samples collected from 373 small animal veterinarians and 120 controls in the general human population for the presence for CaNoV antibodies, and the seropositive rate of the veterinarian group was 22.3% compared to 5.8% in control [16].

The emergence of CaNoVs may be a public health concern because pet animals are an integral part of family life in most industrialized countries, and their close relationship with humans needs to have special attention with potential reservoir of zoonotic agents [17, 18]. Thus, in the present study, we aimed to detect CaNoVs in fecal samples from dogs and to investigate its seroprevalence in the dog population of South Korea.

## Results

### Canine norovirus detection and phylogenetic analysis

**Fig. 1:**
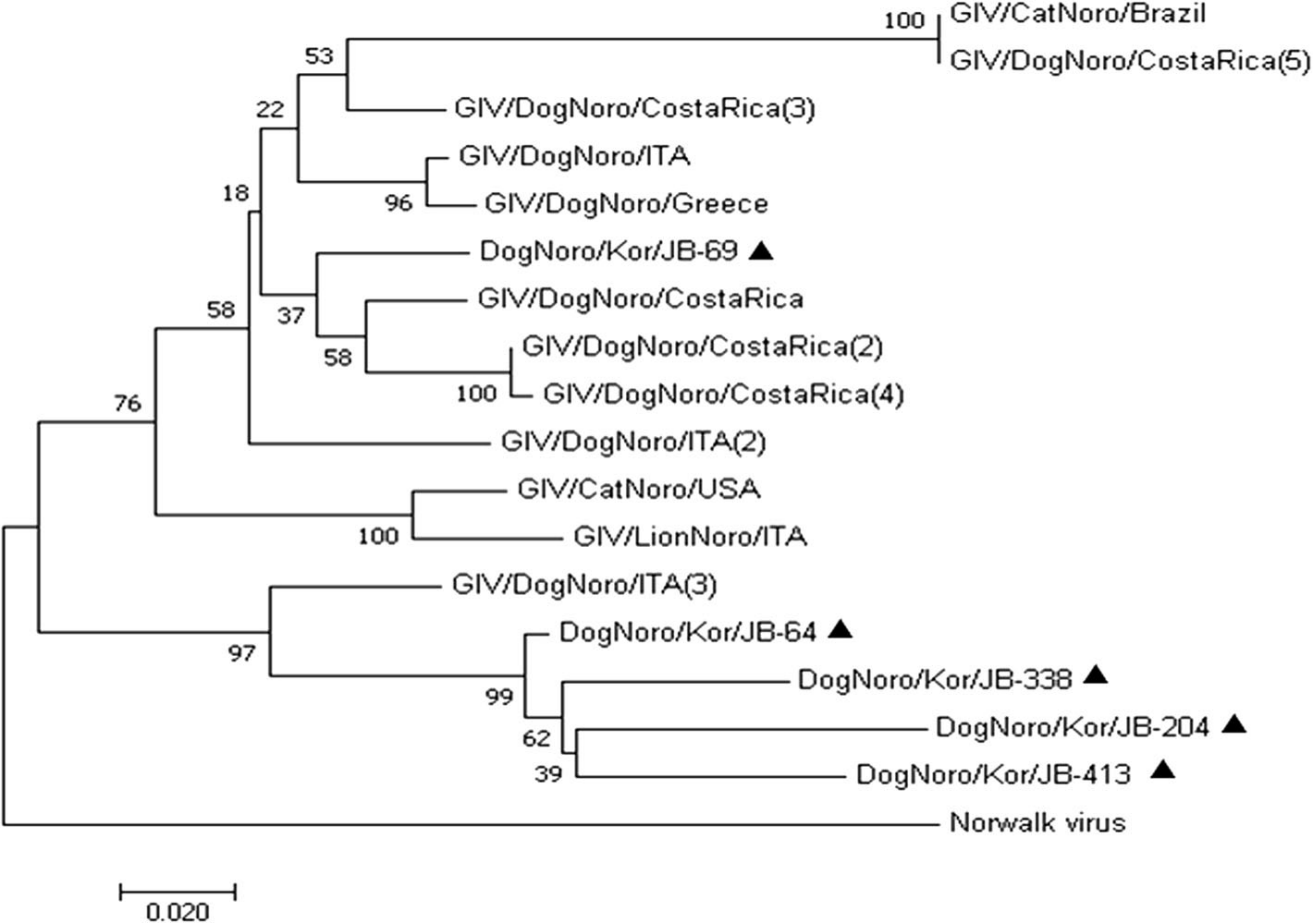
Phylogenetic analysis of CaNoV strains via comparison of nucleotide sequences of partial RdRp region. The analysis was conducted using the MEGA program, and the branches indicate bootstrap values calculated from 1000 bootstrapping replicates. ▲: CaNoV isolates identified in the present study

Fecal samples from 14 (3.1%) of the 459 dog stool samples tested positive for CaNoV. Unfortunately, the completeness of basic data associated with stool samples, including age, sex, breed, and any clinical history of enteric disease varied among the small animal clinics and animal shelters. This precludes further statistically valid analysis of the positive samples. The sequences of partial RdRp region of CaNoVs were analyzed with other CaNoVs sequences from the GenBank database (Fig. 1). The CaNoV isolates in South Korea were classified into the same clade with CaNoV strains detected in Italy and Costa Rica and showed a high nucleotide identity (range 98–100%).

### P domain proteins produced in *E. coli*

**Fig. 2:**
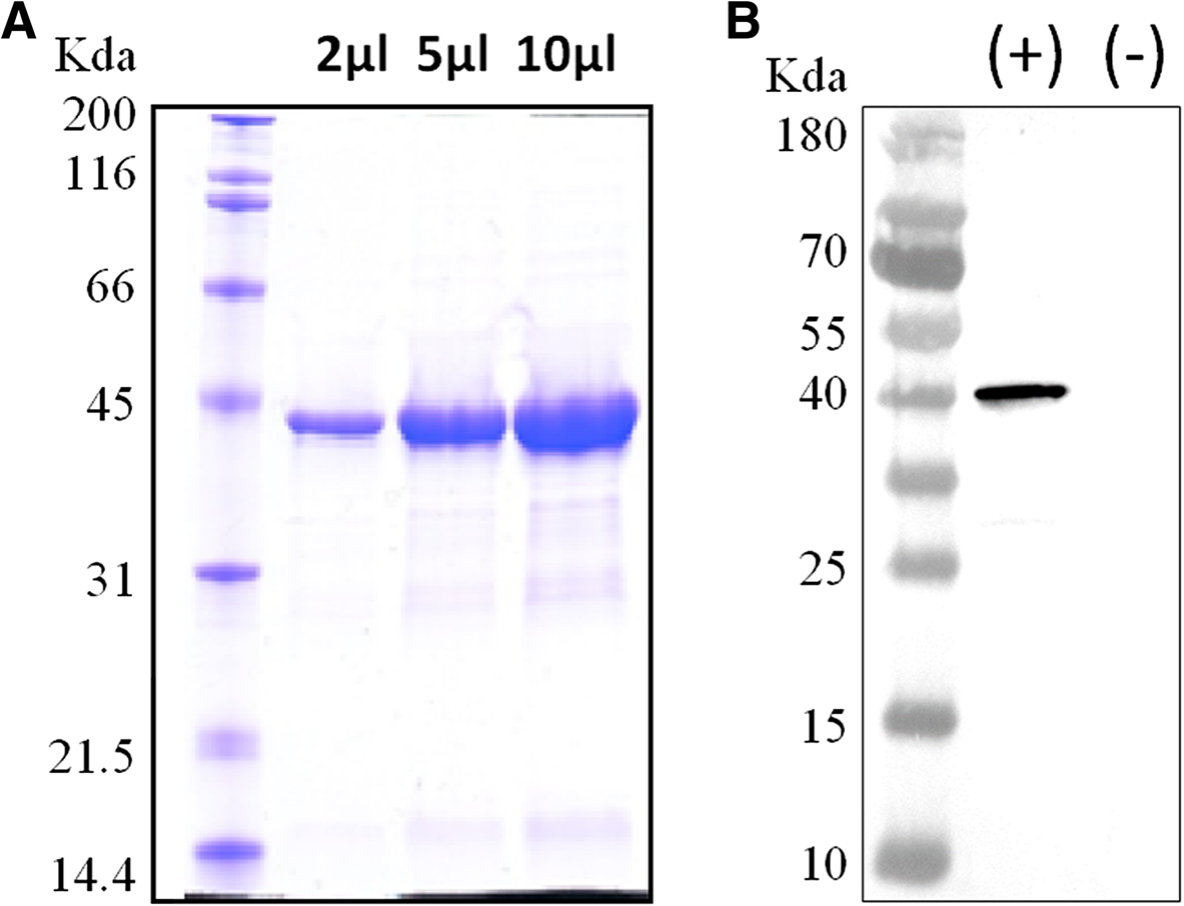
Expression of P domain protein (**a**). Concentration of the purified P domain protein was determined by Bradford assay, and SDS-PAGE gel loaded with 2 μl, 5 μl, and 10 μl of the protein (1 mg/ml) were stained with Coomassie blue. Immunoblotting with CaNoV positive antibody (**b**). The recombinant N proteins were transferred to nitrocellulose membrane, and the membrane was incubated with CaNoV-positive dog serum (lane +) or negative control dog serum (lane -)

The recombinant P domain protein of CaNoV which was successfully expressed as a soluble protein with a C-terminus His6-tag in BL21 Star *E.coli* cells. Expressed P domain proteins were purified by a commercial metal affinity chromatography kit. The purified P domain protein was detected by SDS-PAGE staining with Coomassie brilliant blue which gave a band corresponding to the expected molecular mass (Fig. 2a). To determine the specificity of the expressed P domain protein, western blotting was demonstrated using negative control dog serum and CaNoV positive dog serum which was tested by the ELISA developed in this study (Fig. 2b).

### Seroprevalence

**Fig. 3:**
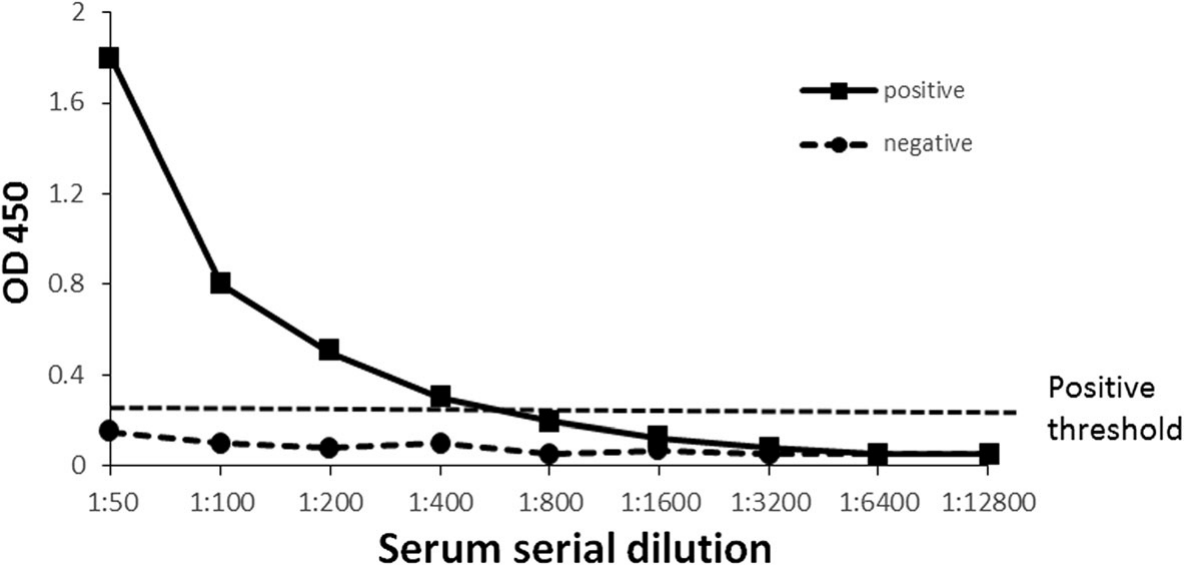
Antibody titers in dog serum samples. Positive serum and negative control serum were diluted from 1:50 to 1:13200 used in the ELISA assay. The corrected OD450 was obtained by subtracting the background signal from the VLP coated well OD450 value. The positive threshold was determined by calculating the mean OD450 of buffer coated wells with the highest serum dilution, plus 3 standard deviations

To determine the seropositivity of serum samples, positive serum and negative control serum were serially diluted two-fold from 1:50 of starting dilution to 1:12,800 and tested by ELISA. A range of titers were identified, and the cut-off threshold was calculated as mean plus three times the standard deviation of the OD450 reading of negative control serum (Fig. 3). Dog serum samples were obtained from small animal clinics in seven provinces; Seoul, Gyeonggi, Gangwon, Chungbuk, Chungnam, Jeonbuk, and Busan, in South Korea and tested by ELISA for antibodies to CaNoV. From the 427 serum samples, 68 (15.9%) tested positive for IgG antibodies against CaNoV. The prevalence varied with respect to the provinces; Seoul (18.0%), Gyeonggi (14.1%), Gangwon (11.6%), Chungbuk (18.9%), Chungnam (11.5%), Jeonbuk (15.4%), and Busan (16.1%).

## Discussion

Recently, dogs were suggested to be potential zoonotic vectors of HuNoV, since HuNoV was detected in stool samples from pet dogs that had been in direct contact with the owner infected with identical HuNoV strain, and the presence of antibodies to HuNoV in dogs was demonstrated in across Europe [15, 17]. In South Korea, the National Agricultural Cooperative Federation reported that companion animal market in South Korea is continuously expanding and is expected to grow at a significant pace in coming years. They forecast that the market will increase from 1 billion U.S. dollars in 2012 to 5.3 billion U.S. dollars in 2020. Regardless of the increased exposure risk to humans with companion animals, there was no previous information concerning the epidemiology of CaNoV in the country. Although a markedly high detection rate of CaNoV in dogs was not demonstrated in the present study, this is the first study describing the detection and prevalence of CaNoV in dogs in South Korea and provides good baseline information for the design of further studies.

CaNoVs detected in the present study were classified into the same cluster with previously reported genogroup GIV CaNoVs in the phylogenetic tree. It was expected that the CaNoV prevalence in fecal samples from the animal shelters would be relatively higher when compared to the samples from small animal clinics as environmental factors such as hygiene reasons or crowded space would be expected to increase transmission between dogs. However, there was no difference in the prevalence between these both dog populations. Seroprevalence of CaNoV was performed by ELISA based on P domain protein, and 15.9% of 427 dog serum samples collected from small animal clinics tested positive for IgG against genogroup GIV CaNoV. These results demonstrate that CaNoV has notably circulated in the dog population in South Korea.

It was previously suggested that CaNoV can easily infect and spread within dog population and its homologous host, and viral characteristics such as incubation and shedding period of this virus resemble to human norovirus [19]. Indeed, dogs contacted with imported dogs from Russia showed diarrhea and diagnosed with CaNoV infection in Portugal, even though the dogs had been vaccinated against major gastroenteritis pathogens [20]. Although the origin of CaNoV in South Korea could not be established in the present study, it is clear that epidemiologic data should be collected from other geographic regions including South Korea.

The P domain of noroviruses comprises a protruded structure of capsid protein which is believed to play the receptor-binding site for virus attachment and viral determinant for immune-response in the host [21, 22]. In the present, the P domain proteins of a genogroup GIV CaNoV were produced in *E. coli* and purified using a Ni-NTA column to develop ELISA, although multiple studies have previously demonstrated to identify antibodies against CaNoV based on virus-like-particle (VLP) antigens produced in a recombinant baculovirus system [15]. The antigenic activities between P domain proteins and VLPs were previously compared, and both antigens showed a similar binding effect and detectable affinity for norovirus specific antibodies [22]. Moreover, the production procedure of the P domain protein in an *E. coli* system is simpler and more cost-effective than in baculovirus expression systems [22, 23].

## Conclusion

This is the first study identifying CaNoV in the South Korean dog population. We successfully developed ELISA based on the P domain protein of CaNoV produced in *E. coli* to detect IgG antibodies and demonstrated the seroprevalence and co-circulation of CaNoV within the dog population using the ELISA.

## Methods

### Sample collection

We collected fecal samples from small animal clinics and animal shelters housing free-roaming dogs in geographically distinct areas in South Korea. The sampling was followed the General Animal Care Guideline as required and approved by the Institutional Animal Care and Use Committee of Chonbuk National University (# CBNU-2018-063). Clinic veterinarians collected only the stools of dogs with consenting owners in their clinics, whereas managers of the animal shelters collected their dog stools. A total of 459 fecal samples were collected from healthy or unhealthy dogs, and then stored at − 20 °C until and during transportation to the laboratory. A total of 427 dog serum samples were collected from small animal clinics in geographically distinct areas of South Korea. Veterinarians with owner’s consent retained the serum remaining after running necessary diagnostic tests on hospitalized dogs. All sera were stored at − 20 °C until transportation to the laboratory.

## RT-PCR and sequence analysis

Fecal samples were diluted 10% (wt/vol) in phosphate-buffered saline (PBS) (pH 7.2). After centrifugation at 8000 g for 5 min, viral nucleic acid was extracted from 140 μl of each supernatant using Viral RNA Isolation Kit (QIAamp Viral RNA Mini Kit, QIAGEN, Hilden, Germany) according to the manufacturer’s instructions. For the detection of CaNoV, reverse transcription PCR (RT-PCR) was performed with the One Step RT-PCR kit (QIAGEN) with previously reported primers: JV102 (5′-TGG GAT TCA ACA CAG CAG AG-3′) and JV103 (5′-TGC GCA ATA GAG TTG ACC TG-3′) [12]. The amplified PCR fragments were purified using the QIAquick Gel Extraction kit (QIAGEN) and sequenced (Bionear, Korea). Analysis of the nucleotide sequences of the CaNoV isolates was performed using BLAST/Align (bl2seq) on the NCBI website and the MEGA (ver.4.1) program. Nucleotide sequences of the isolates were aligned using the ClustalX (ver. 1.81) software, and a phylogenetic tree was constructed by the neighbor-joining method with other CaNoV isolates from GenBank. The phylogenetic distances were determined using bootstrapping with 1000 repeats, and molecular evolutionary analysis was performed using the MEGA (ver.5.0) program.

### Viral protein synthesis

To express the P domain of CaNoV capsid protein, the VP1 gene of a CaNoV (GenBank accession No. GQ443611, NCBI) originated from a dog with diarrhea was synthesized. The P domain (222~570 amino acids) was amplified by PCR using primers: CNVPD-*Nde*I forward (5’-GTTGCCCATATGGAGTCTCGTGTCACCCCTTTTTC-3′) and CNVPD-*BamH*I reverse (5’-CGCGGATCCTTAGGAGCCAGTTCCCACAGGGCTG-3′). The PCR products were cloned into the *Nde*I/*BamH*I restriction site of pET21a (Novagen), which attached a His6-tagged at the C-terminus of the protein. Expression of the P domain protein was performed in BL21 Star *E.coli* cells (Invitrogen). The pET21a-P domain vector-containing *E.coli* cells were incubated with 0.4 mM IPTG (final concentration) to induce protein expression. After 16~18 h of incubation, the cells were harvested via centrifugation. The supernatant was removed, and the cell pellets were suspended in 30 ml of lysis/binding buffer (50 mM Tris-HCl (pH 7.5), 500 mM NaCl, 1 mM phenylmethylsulfonyl fluoride (PMSF), 4 mM 2-mercaptoethanol, 10% glycerol per liter culture volume and sonicated on ice 3x30sec at 60% power. The protein was purified via Ni-NTA Superflow (Qiagen) metal affinity chromatography. The eluate was concentrated and further purified by Superdex 200 size exclusion chromatography (GE Healthcare). The protein was isolated from the final purification step in the twofold PBS buffer and the concentration and purity were determined by Bradford assay. The purified protein and standard BSA protein were separated on 10% SDS-polyacrylamide gels, and then the gel was stained with Coomassie Blue for 1 h at room temperature.

### ELISA procedure

For serology, 96-well polystyrene microtiter plates (Nunc Maxisorp; Fisher Scientific, Waltham, MA, USA) were coated with 50 ng of the purified P domain protein in 0.05 M carbonate-bicarbonate buffer (pH 9.6) overnight at 4°. The plates were blocked with 5% skimmed milk in 0.05% Tween20-PBS (PBS-T) for 1 h at 37 °C and washed three times with PBS-T. Dog serum samples diluted in 1:50 in blocking buffer were incubated for 1 h at 37 °C. Pooled sera from SPF dogs (provided by Green Cross Veterinary Product, Suwon, Korea) was used as a negative control. After three washes with PBS-T, the wells were incubated with goat anti-dog IgG conjugated with horseradish peroxidase (HRP) (Abcam, Cambridge, MA, USA) diluted 1:10,000 in blocking buffer for 1 h at 37 °C. The plates were washed three times with PBS-T, and the antibody reaction was developed with tetramethylbenzidine (TMB) (Sigma-Aldrich), followed by incubation at room temperature for 10 min. The reaction was stopped with 1 N H_2_SO_4_, and the optical density (OD) at 450 nm was assessed in a microplate reader (Model 680; Bio-Rad, Hercules, CA). All sera including negative control serum were tested in duplicate, and they were considered as positive when the corrected OD value was above the mean of the ODs from the negative control wells plus three standard deviations.

### Western blotting

To confirm the specificity of the expressed CaNoV P domain protein, an immunoblotting assay was performed with dog sera tested by the ELISA which was developed in the present study. The protein loaded by SDS-PAGE was transferred onto a nitrocellulose membrane for western blotting. The membrane was blocked in 5% skimmed milk buffer, and then incubated with CaNoV-positive or -negative dog serum at a dilution of 1:500 in TBS-T containing 1% skimmed milk at 4 °C overnight. After washing three times with PBS-T, the membrane was incubated for 1 h with goat anti-dog IgG HRP-conjugated secondary antibodies, and a specific band was detected using ECL reagents (GE Healthcare Life Sciences, Buckinghamshire, UK).

## Abbreviations

CaNoV: Canine noroviruses
HRP: horseradish peroxidase
PBS: phosphate-buffered saline
RdRp: RNA dependent RNA polymerase
TMB: tetramethylbenzidine
